# Differential Toxicity Responses between Hepatopancreas and Gills in *Litopenaeus vannamei* under Chronic Ammonia-N Exposure

**DOI:** 10.3390/ani13243799

**Published:** 2023-12-09

**Authors:** Yuan Zhang, Jianyong Liu, Hongbiao Zhuo, Lanting Lin, Jinyan Li, Shuo Fu, Haiqiong Xue, Haimin Wen, Xiaoxun Zhou, Chaoan Guo, Guangbo Wu

**Affiliations:** 1 College of Fisheries, Guangdong Ocean University, Zhanjiang 524088, China; 13501471667@163.com (Y.Z.); guideng666@gmail.com (H.Z.); kkkkobayashi@163.com (L.L.); jzl02206@163.com (J.L.); 13414945260@163.com (S.F.); xhq1818437@163.com (H.X.); kmjmt010101@163.com (H.W.); 15827297073@139.com (X.Z.); ca32455770@163.com (C.G.); wgb740@126.com (G.W.); 2 Guangdong Provincial Shrimp Breeding and Culture Laboratory, Guangdong Ocean University, Zhanjiang 524088, China

**Keywords:** ammonia-N exposure, *Litopenaeus vannamei*, tissue, differentially expressed genes, mechanisms

## Abstract

**Simple Summary:**

Chronic ammonia-N exposure always exists in realistic cultivation, and it is important to know about the mechanisms of shrimp in response to it and to shorten the breeding cycle. The aim of this study was to investigate the influence of chronic ammonia-N exposure on the growth performance, morphological and physiological alterations, and transcriptome changes of shrimp and to evaluate their ammonia-N resistance after chronic ammonia-N stress. Results showed that chronic ammonia-N exposure affected growth, caused significant structural damage and vacuolation in hepatopancreas and gill tissues, and changed related antioxidant enzyme activities. The gills maintained cellular homeostasis mainly through the high expression of cytoskeleton and transcription genes, whereas the hepatopancreas down-regulated related genes in the ribosome, proteasome, and spliceosome pathways. These genes and pathways are important in the biosynthesis and transformation of living organisms. In addition, both tissues maintained organismal growth primarily through lipid metabolism, which may serve as an effective strategy for ammonia-N resistance in *L. vannamei*.

**Abstract:**

Ammonia nitrogen is one of the main toxic substances in aquatic cultivation environments. Chronic exposure to excessive amounts of ammonia-N creates toxic consequences, retarding the growth of aquatic organisms. This study investigated the growth performance, morphological and physiological alterations, and transcriptome changes in the hepatopancreas and gills of white shrimp *Litopenaeus vannamei*. The results showed that there was no significant difference in the survival rate (*p* > 0.05), whereas growth performance was reduced significantly in the treated groups compared to the control groups (*p* < 0.05). Significant structural damage and vacuolation occurred in hepatopancreas and gill tissues in the treated groups. Superoxide dismutase (SOD) activity and Na^+^/K^+^-ATPase content were significantly increased by chronic ammonia-N exposure in the two tissue groups. In addition, catalase (CAT) activity and malondialdehyde (MDA) levels were significantly altered in the hepatopancreas groups (*p* < 0.05), whereas no differences were observed in the gill groups (*p* > 0.05). There were 890 and 1572 differentially expressed genes identified in the hepatopancreas (treated versus control groups) and gills (treated versus control groups), respectively, of *L. vannamei* under chronic ammonia-N exposure. Functional enrichment analysis revealed associations with oxidative stress, protein synthesis, lipid metabolism, and different serine proteases. The gills maintained cellular homeostasis mainly through high expression of cytoskeleton and transcription genes, whereas the hepatopancreas down-regulated related genes in the ribosome, proteasome, and spliceosome pathways. These genes and pathways are important in the biosynthesis and transformation of living organisms. In addition, both tissues maintained organismal growth primarily through lipid metabolism, which may serve as an effective strategy for ammonia-N resistance in *L. vannamei*. These results provided a new perspective in understanding the mechanisms of ammonia-N resistance in crustaceans.

## 1. Introduction

The Pacific white shrimp, *Litopenaeus vannamei*, accounts for ~80% of the total shrimp market because of its rapid growth rate and tolerance to a wide range of salinities and temperatures [1]. However, the rapid expansion in shrimp farming has created some unavoidable problems, such as disease outbreaks, inadequate culture technologies, and variable and deteriorated aquaculture environments, which accompany them on account of the rapid expansion in shrimp farming [2]. The yield and quality of shrimp are affected by the degradation of culture water environmental factors [3].

The most common dissolved inorganic nitrogen ions include ammonia-N, nitrite-N, and nitrate-N, which are interrelated via the nitrification cycle [4]. A previous study reported that ammonia-N was usually converted to nitrite-N and then to nitrate-N by aerobic chemoautotrophic bacteria, such as Nitrosomonas and Nitrobacter [5], and finally to N_2_ via aerobic denitrification [6]. Ammonia is an essential and low-life-sustaining nutrient for marine phytoplankton [7]. In some fish species, low concentrations of exogenous ammonia induce protein synthesis and body growth [8,9]. However, excessive ammonia cannot be fully absorbed and is excreted by the shrimp. Ammonia accumulates in the water because of the ammonification originating from organic detritus, such as excess feed and feces in the intensive culture systems [10]. High concentrations of ammonia lead to the pollution of aquatic water, reducing growth rates [11], affecting osmoregulation [2], causing cellular toxicity [12], increasing the susceptibility of aquatic organisms to infection [13], and even resulting in mortality. Additionally, ammonia affects the profusion and physiological conditions of different aquatic animals; among them, the decapod crustaceans are particularly vulnerable to it due to the toxic effect of ammonia, which can influence survival, metabolism, immune function, and oxidative stress [5,14,15].

Oxidative stress in the hepatopancreas of shrimp at a total ammonia concentration of 20 mg/L can lead to endoplasmic reticulum stress and apoptosis [16]. Liu et al. reported that organisms are able to regulate and resolve the excess reactive oxygen species (ROS) that are induced by chronic exposure to Pb (during a 28-day indoor exposure study) at low concentrations in the hepatopancreas of *Macrobrachium nipponensis* [17]. In aquatic organisms, excessive ROS generated by accumulated ammonia in cultivation water induce oxidative stress and lead to toxic effects, such as the loss of cell function and even apoptosis [14]. Enzymatic and nonenzymatic mechanisms belong to self-protection processes, which are established by organisms to eliminate ROS-mediated damage [18]. A previous study suggested that high ammonia-N exposure induced not only oxidative stress but also ER stress [19]. Chronic exposure to ammonia-N reduces the shrimp’s food intake and disrupts energy metabolism by lowering body lipids and increasing carbohydrate content, leading to the slower growth of the shrimp [20]. Additionally, osmoregulation and ammonia excretion are also affected, and this changes Na^+^/K^+^-ATPase activity [21].

In aquatic animals, the hepatopancreas and gills are vital tissues during ammonia-N exposure [22]. The hepatopancreas is associated with accumulation and detoxification [23], and the gill tissue is the first tissue to respond to negative environmental factors [24], acting as a biomarker for aquatic toxicology. A previous study revealed that specific enzymes and proteins in the gills coordinate with each other to realize different functions, such as ion transport, acid–base balance, and osmotic balance [25]. At the same time, Zhang et al. also pointed out that the hepatopancreas could reserve lipids and synthesize and secrete digestive enzymes [25]. Li et al. suggested that chronic exposure to ammonia-N damaged the structure and disturbed the lipid metabolism of the hepatopancreas in shrimp [5]. Zhu et al. reported that the hepatopancreas contained superoxide dismutase (SOD) and catalase (CAT), which are related to physiological metabolism and immune reactions in the antioxidant defense system [26]. In addition, Zhang et al. reported a comparative transcriptomic analysis of three tissues to unveil a network of energy reallocation, which suggested that the hepatopancreas and gills cooperate with each other rather than muscles in response to heat stress [27]. These findings indicated that the analysis of the histological structure, physiological indicators, and molecular response of the hepatopancreas and gills may explain various stress mechanisms of crustaceans. However, limited data exist on the response of shrimp tissues to ammonia-N exposure.

In this study, the effects of exposure to 0 mg/L (control) and 8 mg/L (NH_4_CL solution) ammonia-N for 28 days were analyzed through growth performance, morphological, physiological, and transcriptional changes. We also analyzed changes in the physiological indices and the histological structure and characteristics of gene expression in the hepatopancreas and gills. Exploring the mechanism of ammonia-N tolerance in crustaceans will further enable shortening of the breeding cycle of new varieties suitable for this environment. Furthermore, the current study will supply us with a theoretical basis for decreasing the adverse effects and will increase our knowledge about the risk of ammonia-N stress in aquaculture.

## 2. Materials and Methods

### 2.1. Ethics Statement

All animal experiments were carried out following the guidelines and approvals of the Institutional Animal Care and Use Committee of Guangdong Ocean University (Zhanjiang, China).

### 2.2. Experimental Animals

**Figure 1:**
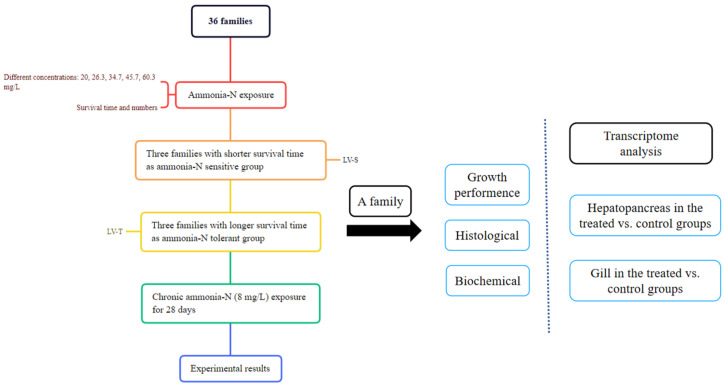
Schematic presentation of experimental shrimp from 36 families.

This research used *L. vannamei* shrimp obtained from 36 full-sib families, which were produced at the Zhanjiang Guoxing Aquatic Technology Co., Ltd. (Zhanjiang, China), following the same methods as described previously [28]. To determine ammonia-N tolerance, an acute ammonia-N stress test following a logarithmic spacing of concentration (20, 26.3, 34.7, 45.7, 60.3 mg/L) was conducted with juveniles from 36 families. The pre-experiment was adjusted and carried out to detect the half-lethal time under the experimental conditions according to the ammonia doses reported previously [29]. The results showed that the survival time changed among these families under acute ammonia-N exposure compared to the control groups. We selected ammonia-tolerant (LV-T) and sensitive (LV-S) groups according to the duration of survival time. We investigated the difference in ammonia-N response and growth performance between groups. Shrimp from the same LV-T families were selected based on survival time for the control and chronic cultivation (Figure 1). The ammonia solution used to modify the ammonia-N content was prepared using NH_4_Cl (Xilong Scientific Co., Ltd., Shantou, China). All shrimp were not fed during the experiment. Shrimp were acclimated for one week before the experiment in a cement pool (2 × 2 × 1.5 m), which contained sand-filtered seawater (25.0–28.0 °C, 28–30 ppt, pH 7.9–8.0), had a photoperiod of 12 h light and 12 h darkness, and was fitted with an air pump. They were fed four times each day (7:00, 11:30, 16:00, and 23:00) with standard feed (Quanxing Aquatic Feed Co., Ltd., Foshan, China) and vitamin-C soluble powder (Hebei Hao Hai Biologicag Co., Ltd., Qinhuangdao, China) mixed evenly, and waste was removed from the bottom of the plastic frame. One third of the total aquaculture water was replaced daily. The hepatopancreas and gills of these individuals were obtained for experiments.

### 2.3. Experimental Treatments

After acclimation, the shrimp were classified at random into test (with ammonia solution concentration of 8 mg/L) and control groups (normal seawater without extra ammonia solution addition) in a cement pool (15 m^3^), respectively, with three baskets of 40 shrimp in the two groups. Chronic exposure concentrations 8 mg/L for 28 days were based on the simple pre-experiment, reported environmental concentrations, and related studies. Lu et al. reported that the treated group was cultured under an ammonia-N concentration of 10 mg/L [30]. Dong et al. suggested that the survival rate of the highest concentration group (10 mg/L) was significantly lower than that of the control group (*p* < 0.05), whereas no significant difference occurred between other experimental groups and the control group (*p* > 0.05) [31]. The seawater temperature was maintained at 25.0–28.0 °C during the experiment, and a multiparameter water quality detector (Xiamen PanTian BioTech Co., Ltd., Xiamen, China) was used to measure dissolved oxygen (DO), pH, and total ammonia each day. In addition, 10–15% of the volume of seawater according to water quality and color was exchanged every day with ammonia solution or natural seawater to maintain a stable ammonia-N concentration and conditions in the treated and control groups, respectively. Cement pools were cleaned of feces daily using a sump pump (Goovssy, Xiamen, China). Whenever a shrimp showed loss of equilibrium (LOE) and was unable to recover dorso-ventral orientation within 5 s of external stimulation, the corresponding time was recorded.

### 2.4. Sampling and Calculations

During the experimental period, the water temperature, pH, salinity, and ammonia-N concentration of the treated and control groups were regularly measured four times per day (Table S1). At the same time, the shrimp were monitored and removed, and survival time was recorded throughout the experiment (Table S2). The shrimp abstained from food for 24 h before sampling after 28 days of exposure. Body length (BL) was the distance from the base of the eye stalk to the tip of the telson. BL was measured using an electronic vernier caliper, accurate to 0.01 mm. We used an electronic balance to measure body weight (BW), accurate to 0.01 g. Water on the shells of freshly collected shrimp was absorbed using gauze prior to taking BL/BW measurements.

The hepatopancreas and gills of three individuals were taken from each group, cut into pieces immediately after dissection, put into RNAhold (Trans, Beijing, China) reagent overnight, and then stored at −80 °C for experiments. These samples were delivered to Novogene Biotech Co., Ltd. (Tianjin, China) for transcriptome analysis and qPCR validation. The two tissues from the same number of shrimp (*N* = 18) were used to assess enzyme activities and were quickly frozen with liquid nitrogen; they were then stored at −80 °C. To observe histological change, tissues from shrimp (*N* = 18) were fixed in 4% paraformaldehyde fixing solution (Biosharp, Beijing, China) and examined. We used the following mathematical equations to calculate various growth parameters:

S (%) = (S_f_/S_i_) × 100%

where S, S_f_, and S_i_ represent the survival rate, the numbers of surviving individuals at the end of the experiment, and the total number of individuals at the start of this stage, respectively.

BL (cm) = B_1_ − B_0_ WG (%) = (WG_1_ − WG_0_)/WG_0_ × 100% SGR (% d^−1^) = [(WG_1_ − WG_0_)]/(T_1_ − T_0_) × 100%

where BL/WG is the growth of body length/weight gain; B_0_/WG_0_ and B_1_/WG_1_ are the data at the beginning/end of the experimental stage, respectively; and T_0_ and T_1_ are the times of the beginning/end of the experiment. SGR is the specific growth rate [32] and is the average daily growth rate of the shrimp during the experiment.

### 2.5. Histological Examination of the Two Tissues

The hepatopancreas and gills of a shrimp from each plastic frame in the experimental and control groups of the two tissues were dissected from the cephalothorax. The two tissues were fixed with 4% paraformaldehyde for fixation and stored at 4 °C. Paraffin sections a thickness of about 5 μm were prepared following the standard H&E staining processes using Servicebio (Wuhan, China) and examined under a light microscope (SDPTOP RX50, Zhejiang, China) to observe the histopathological changes.

### 2.6. Assessment of Enzyme Activities

The deionized water was used to grind the two tissues at 4 °C. The supernatant was gathered from the homogenate after centrifuging at 3000 rpm for 20 min at 4 °C for use in biochemical determination. The activities of antioxidant enzyme, such as superoxide dismutase (SOD), malondialdehyde (MDA), catalase (CAT), and Na^+^/K^+^-ATPase in the two tissues of shrimp, were measured using ELISA kits from Enzyme-linked Biotechnology Co., Ltd. (Shanghai, China) according to the manufacturer’s instructions.

### 2.7. Transcriptome Sequencing

Total RNA was extracted from the samples using Trizol reagent (Invitrogen Life Technologies, Carlsbad, CA, USA) following the manufacturer’s instructions. The extracted RNA was treated with RNase-free water, which was prepared with 75% ethanol to remove any potential contamination from DNA molecules. Subsequently, the RNA Nano 6000 assay kit of the Bioanalyzer 2100 system (Agilent Technologies, Santa Clara, CA, USA) was used to assess the total amount and integrity of the RNA following the manufacturer’s protocol.

Briefly, the poly-T oligo-attached magnetic beads were used to purify total RNA to mRNA, and divalent cations were used to fragmentate under elevated temperature in First Strand Synthesis Reaction Buffer (5×). The first strand cDNA was synthesized by M-MuLV reverse transcriptase and random hexamer primer, and the RNA was degraded using RNaseH. DNA Polymerase I and dNTP were used to synthesize the second-strand cDNA. The remaining overhangs were translated into blunt ends through exonuclease/polymerase activities. An adapter with a hairpin loop structure was ready for hybridization after the adenylation of the 3′ ends of DNA fragments. The AMPure XP system (Beckman Coulter, Beverly, CA, USA) was used for the selection of cDNA fragments with 370–420 bp. Eventually, AMPure XP beads were used to purify the PCR product following PCR amplification so as to acquire the library.

The library was tested to ensure the quality. After construction of the library, quantification was first performed using a Qubit2.0 fluorometer; dilution to 1.5 ng/µL was then performed. The insertion fragment size of the library was measured using an Agilent 2100 bioanalyzer. After the insertion fragment size was as expected, the effective concentration of the library was accurately quantified using qRT-PCR (the effective concentration of the library was higher than 2 nM) to ensure the quality of the library. When the libraries were qualified, they were sequenced with an Illumina NovaSeq 6000 to produce end reads of 150 bp. The raw data were stored in the National Center Biotechnology Information (NCBI) Sequence Read Archive (SRA) with bioproject number PRJNA993130.

### 2.8. Transcriptome Analysis

CASAVA base recognition was used to image data from high-throughput sequencing measurements into sequence data (readings). In-house perl scripts were used to process raw data/reads into the fasta format. Clean data/reads were obtained at this stage by removing reads containing adapters, low-quality reads, and N bases, and then the Q20, Q30, and GC contents were calculated. Q20 means that every 100 bp of sequencing reading may contain an error, with a call accuracy of 99%. Similarly, a sequencing quality reaches Q30 is perfect and represents an accuracy of 99.90%, with no errors or ambiguities. HISAT2 (version 2.0.5) software was used to map the filtered reads with high-quality clean data from the *L. vannamei* reference genome (NCBI accession No. PRJNA927338). FeatureCounts v1.5.0-p3, using the default parameters, was used to count the readings mapped to every gene. In addition, the number of transcript fragments per kilobase (FPKM) per million mapping readings of every gene was computed according to the gene length and readings mapped to it. To determine the effects of long-term ammonia-N exposure (8 mg/L) and normal aquatic concentrations (less than 0.25 mg/L) on the hepatopancreas and gill of shrimp, differentially expressed genes (DEGs) were analyzed in groups using the DESeq2 R package (1.20.0). In the study, |log2 (fold change)| ≥ 1 and false discovery rate (FDR) < 0.05 were used to represent the DEGs of the transcriptomic differences between the two groups. DEGs analyzed using the tools including GO (http://www.geneontology.org/) and KEGG (https://www.genome.jp/kegg/) pathway enrichment analysis, which accessed on 1 November 2022 and 17 December 2019, respectively, to determine the significant pathways (*p* ≤ 0.05) of the regulatory genes. 

### 2.9. Data Validation via qPCR

To validate the gene expression profiles and an accuracy of the RNA-seq data analysis, samples from the hepatopancreas groups (with three replicates), were analyzed by qPCR. Eight gene-specific primers for DEGs were designed, using Primer5 software (Premier Biosoft International, Palo Alto, CA, USA) (Table S3) for qPCR amplification, according to the *L. vannamei* genome and assembled transcriptome data. The qPCR testing was performed following the manufacturer’s instructions, using a CFX96 Real-Time PCR detection system (Bio-Rad, Hercules, CA, USA) and Perfect Start Green qPCR SuperMix (TransGen). The qPCR was performed as described in a previous study by Liang et al. [33]. The 2^−ΔΔCt^ method was used for evaluating the relative genes expression levels through the cycle thresholds (Cq), which were transformed from fluorescence information, with *β*-*actin* (GenBank accession No. XM_027364953.1) and served as an internal reference gene for normalization.

### 2.10. Data Analysis

DESeq2 R package (1.20.0), |log2 (fold change)| ≥ 1 and false discovery rate (FDR) < 0.05 were used to determine the DEGs. Growth and qPCR data of the study were analyzed with a T test in SPSS 23.0 (SPSS Inc., Chicago, IL, USA). The T test was used to determine the significant differences between genes after the normality of distribution and homogeneity of variance. Excel 2016 (Microsoft, Redmond, WA, USA), GraphPad Prism 8.0.2 (La Jolla, CA, USA), and Origin 18 (OriginLab, Northampton County, MA, USA) were used to analyze all statistical data and generate images. The results were expressed as mean ± standard error (SD). The differences were statistically significant at *p* < 0.05.

## 3. Results

### 3.1. Survival Rate and Growth Performance

We observed abnormal behavior in the exposed shrimp, such as reduction in motility, decrease in feed intake, and erratic swimming. There were no behavioral alterations during the experiment in the control groups, were no significant differences in survival rate (*p* > 0.05), whereas BL, WG, and SGR were significantly reduced (*p* < 0.05) between the control and experimental groups (Table 1). In addition, the BL, WG, and SGR decreased by 27.96%, 43.87%, and 37.02%, respectively, compared to the control groups.

### 3.2. Histological Analysis of the Two Tissues

**Figure 2:**
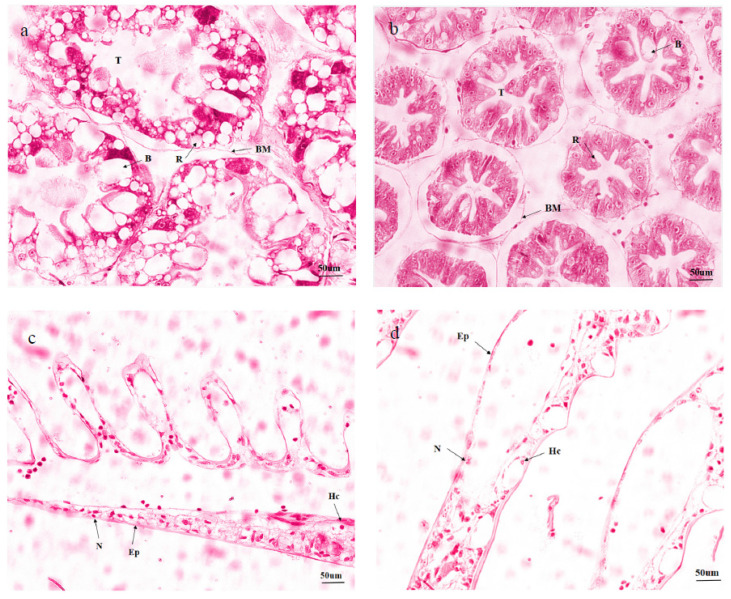
Histological examination of hepatopancreas and gills of *L. vannamei* subjected to ammonia-N for 28 days with the treated group (**a**,**c**) and with the control group (**b**,**d**). Note: R: storage cells (R-cell); B: secretory cells (B-cell); T: the star-shaped polygonal structure of the hepatopancreatic lumen. BM: basement membrane; Ep: epithelial cell; N: epithelial nucleus; Hc: hemocytes. Stained using hematoxylin and eosin (H&E), 400×.

The hepatopancreas and gill tissues showed significant histological changes after 28 days of ammonia-N stress (Figure 2). The damage reduced storage cells (R cells), increased secretory cells (B cells), and distorted or disappeared the star-shaped polygonal structures of the lumen (Figure 2a). At this time, the boundaries of the hepatopancreatic tubules were vague, with significantly expanded volume, and some cells were disassociated (Figure 2a). The boundaries of hepatopancreatic tubules were distinct, with complete basement membranes and arranged hepatocytes in the control group (Figure 2b). The shrimps exposed to 0 mg/L ammonia-N had normal gill epithelium arrangement and cell contour without cell vacuolation or congestion (Figure 2d). The gills of *L. vannamei* subjected to 8 mg/L ammonia-N exposure displayed damaged histological structure compared to the control groups. The gill filaments began to deform and swell, quantities of hemocytes increased, and a large number of epithelial cells shed compared to the control groups (Figure 2c).

### 3.3. Enzyme Activities of the Two Tissues

**Figure 3:**
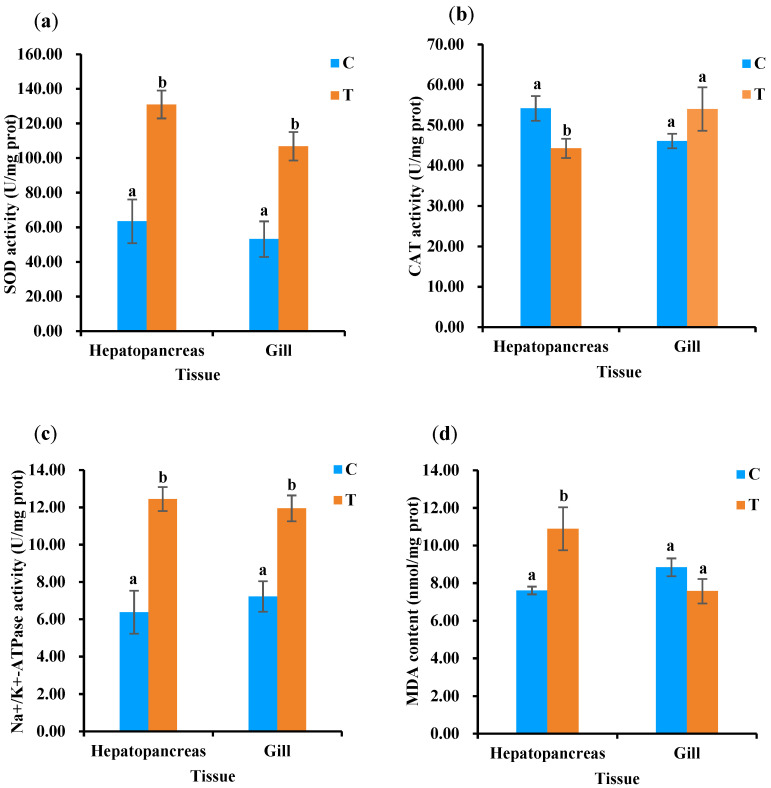
Effects of chronic ammonia-N on the nonspecific immune indices in the hepatopancreas and gill of *L. vannamei*. (**a**) SOD activity, (**b**) CAT activity, (**c**) Na^+^/K^+^-ATPase activity, (**d**) MDA content. Note: different letters indicate significant differences between treated (T) and control (C) groups (*p* < 0.05).

The changes in the antioxidant statuses of the two tissues of *L. vannamei* between chronic-ammonia-exposed and control groups are showed in Figure 3. The results found that SOD activity significantly increased (*p* < 0.05) between the treated and control groups in the two tissues (Figure 3a). CAT activity had no significant differences in the gills (*p* > 0.05), whereas it was significantly decreased (*p* < 0.05) in the hepatopancreas in the 28-day exposure study (Figure 3b). The activity of Na^+^/K^+^-ATPase was significantly increased (*p* < 0.05) in the two tissues (Figure 3c). Moreover, MDA content, which represents the degree of oxidative stress in the tissues, was significantly increased in the hepatopancreas compared to the control groups (*p* < 0.05), without significance in the gill tissue (Figure 3d).

### 3.4. Assembly, Read Mapping, and Sequence Alignment

The low-quality sequences were removed following RNA-seq analysis of hepatopancreas and gill samples from hepatopancreas and gill groups, respectively. The sequencing data with Q20, Q30, and GC contents of about 98%, 93%, and 49%, respectively, in the hepatopancreas in treated vs. control groups were considered reliable. Similarly, in the gill in treated vs. control groups, the Q20, Q30, and GC contents were about 97%, 92%, and 43%, respectively (Table S4). Compared to the reference genome, there were 266, 212, and 458 valid reads, respectively, and 85.79–89.94% of them were mapped to the genome in the hepatopancreas in treated vs. control groups. In the gill in treated vs. control groups, there were 127, 371, and 814 valid reads, respectively, and 85.79–89.94% of them were mapped to the genome (Table S5).

### 3.5. DEG Analysis in the Two Tissues under Ammonia-N Exposure

**Figure 4:**
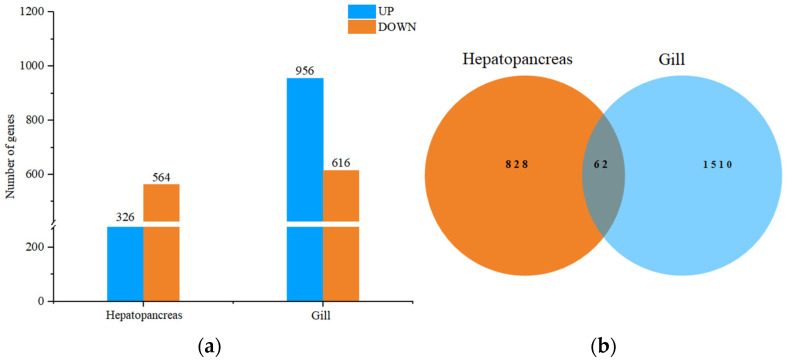
The expression profiles of differentially expressed genes (DEGs). Note: The number of DEGs in each comparison group (**a**). Venn diagram of DEGs between adjacent and nonadjacent pairwise comparison groups (**b**). Orange and blue circles represent genes expressed between hepatopancreas and gill groups, respectively (|log2(FC)| ≥ 1 and FDR < 0.05).

The transcriptome data of the two tissues in the two groups of *L. vannamei* were analyzed to identify the DEGs. Altogether, 890 (326 up-regulated and 564 down-regulated) and 1572 (956 up-regulated and 616 down-regulated) candidate genes were significantly differentially expressed between the experimental and control groups of the two tissues, respectively (Figure 4a). Sixty-two overlapping DEGs were screened with Venn diagrams in order to explore logical association between the two tissues (Figure 4b). The top 10 up-regulated, down-regulated, and overlapping DEGs are displayed in Table 2 with descriptions. A total of 62 DEGs in both tissues—6.97% and 3.94%, respectively—confirmed that the response of different tissues to ammonia-N stress was tissue-specific. Notably, the top terms of DEGs that were differentially expressed after chronic ammonia-N stress included serine-type endopeptidase activity, oxidoreductase activity, and serine hydrolase activity. The cytoskeleton-associated genes were listed in Table 3.

### 3.6. GO and KEGG Enrichment Analysis

**Figure 5:**
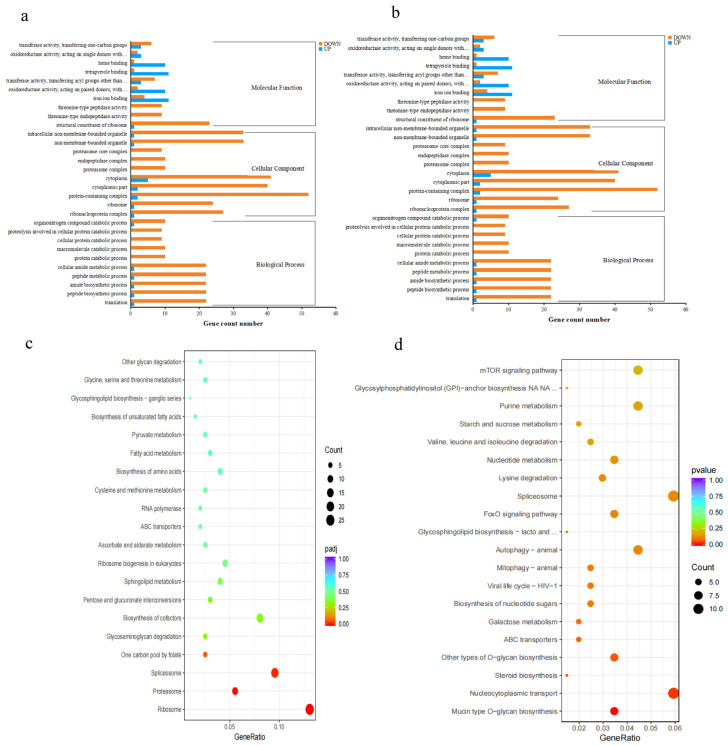
Functional annotation analysis of DEGs under long-term ammonia-N stress. GO enrichment analysis of DEGs between the control and treated groups in hepatopancreas (**a**) and gills (**b**). KEGG pathway enrichment analysis of DEGs between the control and treated groups in hepatopancreas (**c**) and gills (**d**).

The distribution of DEGs’ enriched functions via GO analysis between the ammonia-treated and control groups was generally consistent (Figure 5). The DEGs from GO enrichment analysis were assigned to 3 major functional classes—biological process (BP), cellular component (CC), and molecular function (MF)—and 107 subcategories between treated vs. control groups in hepatopancreas (Figure 5a). BP-enriched DEGs were associated with 47 significantly terms, including translation, peptide biosynthetic process, amide biosynthetic process, peptide metabolic process, cellular amide metabolic process, and so on. Another 31 highly enriched terms were classified as CC, including ribonucleoprotein complex, ribosome, protein-containing complex, cytoplasmic part, cytoplasm, proteasome complex, and endopeptidase complex. The remaining 29 significantly enriched terms belonged to MF. Among the nine pathways, ribosome and ribosome biogenesis were significantly affected by ammonia-N exposure, with most genes being mostly down-regulated, except the first gene of the ribosome pathway in our study (Table 4).

In the hepatopancreas in treated vs. control groups, there were nine significantly enriched terms classified as BP, including the cellular carbohydrate metabolic process, protein de-ubiquitination, protein modification via small protein removal, and the nucleobase-containing compound biosynthetic process (Figure 5b). The cell junction (GO:0030054) was the only one highly ranked in the CC category. The MF category included the remaining 23 significantly enriched terms. However, there were no significantly enriched overlap terms classified in the three functional classes between the transcriptome analysis of the two tissues. It is important to explore gene interactions because of their value in biological functions. To understand potential gene interactions under chronic ammonia-N stress, all DEGs were searched and assigned to nine and three pathways in hepatopancreas and gills, respectively, and the top 20 enrichment pathways shown in Figure 5. The analysis revealed that the DEGs in the groups were significantly enriched in the following pathways: ribosome, proteasome, spliceosome, and one carbon pool by folate; glycosaminoglycan degradation, glycosaminoglycan degradation, biosynthesis of cofactors, pentose and glucuronate interconversions, sphingolipid metabolism, and ribosome biogenesis in eukaryotes (Figure 5c). In the gills in treated vs. control groups, mucin-type O-glycan biosynthesis, nucleocytoplasmic transport, and steroid biosynthesis were the significantly enriched pathways of the DEGs (Figure 5d). Table 4 lists the major enriched KEGG pathways associated with the DEGs. In addition, the two tissues maintain shrimp growth primarily through lipid metabolism (Table 5).

### 3.7. Validation of RNA-Seq Data by qPCR

**Figure 6:**
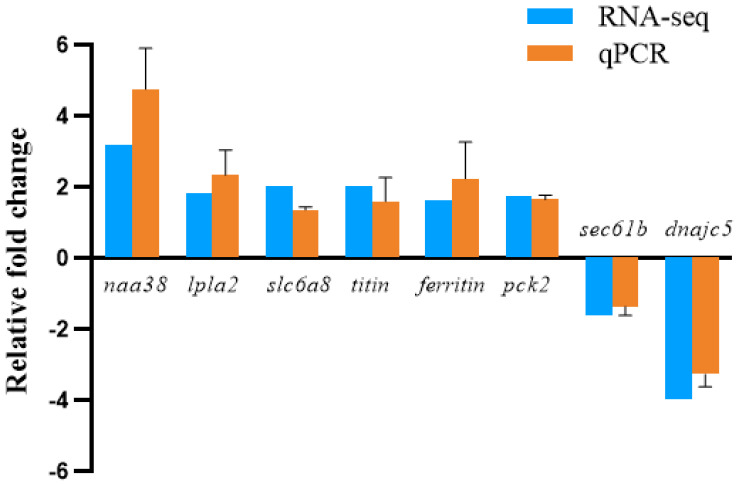
Eight validated genes expression levels in the hepatopancreas groups at 28 days.

Eight DEGs were randomly selected from RNA-seq data for qPCR analysis to verify the accuracy of the Illumina sequencing results (Figure 6). The eight DEGs (six up-regulated and two down-regulated) related to hepatopancreas transcriptome data were lysozyme-C-like (*dnajc5*), lysosomal phospholipase A2 (*lpla2*), sodium- and chloride-dependent GABA-transporter-2-like (*slc6a8*), titin, ferritin-subunit-like, Penaeidin-2b (*naa38*), protein-transport-protein-sec61-subunit-beta-like (*sec61b*), and phosphoenolpyruvate carboxykinase (*pck2*). Overall, the qPCR analysis confirmed that the RNA-seq data were consistent with the expression patterns of the DEGs.

## 4. Discussion

### 4.1. Survival Rate and Growth Performance under Ammonia-N Exposure

Previous studies have shown that excess ammonia increased susceptivity to pathogens, thereby inhibiting growth, reducing osmoregulation, increasing molting frequency, and even leading to high mortality in shrimp [34]. Non-ionic ammonia exposure at 0.54 mg/L in the hepatopancreas of *M. rosenbergii* significantly reduced the survival rate, whereas it had no obvious effect on growth compared to other exposure concentrations (0, 0.108, 0.216, 0.324, or 0.54 mg/L) in a 20-day experiment [31]. Similarly, the survival rate has no significance in blunt snout bream after long-term ammonia-N exposure [22]. The research revealed that the BL, WG, and SGR of shrimp decreased significantly with long-term ammonia-N stress, which affected the growth performance (*p* < 0.05). This result is consistent with the reported long-term experiment with a higher temperature in summer [35]. Chronic ammonia-N exposure could affect the food intake and then disturb this growth [20], which explains why the growth performance of the shrimp differed compared from that of the control groups. The sustained ammonia-N stress decreased the growth of shrimp, which extended the cultivation culture cycle (generally about four months) and increased cost. Aquatic animals may be subjected to potential toxic interactions with nitrate-N because wastewater components often contain more than one nitrogen-containing waste [36]. Moreover, three pollutants in the culture water often exceed those found in nature [37]. In summary, it has been reported that many aquatic species showed a reduction after long-term exposure to ammonia-N, which demonstrates the importance of the cultivation environment. In our study, there was no significant difference in the cumulative death rate after long-term stress of 28 days, indicating that ammonia-N with 8 mg/L concentration was in the adaptive range of the shrimp and could affect their growth.

### 4.2. Tissue Damage and Physiological Responses

In this study, the ammonia-treated groups of the two tissues showed obvious histopathological changes in the irregular appearance of tubular structure after four weeks of rearing compared to the control groups. R cells have many small lipid-filled vacuoles for the synthesis of lipoproteins and their export to other organs, heavy metal detoxification, and uric acid excretion. B cells are regarded as an important lipid reserve in tissues because of their functions of producing, secreting, and recycling fat emulsifiers [38]. In addition, they take charge of the accumulation and transport nutrients, which are also sensitive to toxic substances and other stresses [39]. They are important components belonging to the hepatopancreas. Compared to the control groups, a common feature in the hepatopancreas was moderate-to-high vacuolation in R cells. Similar results have been found with *Marsupenaeus japonicus* [40] and *M. nipponense* [38]. This suggests that ammonia-N caused damage to the hepatopancreas structure, leading to a reduction in R cells, and then secretion and exretion of toxic substances through B cells, resulting in an increase in B cells. The previous study reported that the infestation by *Probopyrus bithynis* affected the structure of gills in *M. amazonicum,* including swelling, elevation of epithelial cell hyperplasia, and hemocyte accumulation [41]. Guo et al. observed that the structure change in gills was different at various points—cellular tumefaction and laminar epithelial thickening in particular [42]. Similarly, the symptomatology of the study was consistent with this when the shrimp were exposed to 15 mg/L ammonia-N for 28 days [43]. These studies suggest that the histopathological changes may reflect an adaptation to the stress caused by chronic ammonia-N and lead to thickening of the epithelium, which increases the distance between the internal and external media.

Ammonia in aquaculture water will accumulate and produce excess ROS in tissues, which induces oxidative stress, creates toxic effects on aquatic organisms, and leads to functional defects and even apoptosis [14]. Antioxidant enzymes are produced by organisms fighting against ROS and balancing the homeostasis between oxidants and antioxidants in living organisms. In the study, levels of SOD were increased between the experimental and control groups (*p* < 0.05), and CAT was increased just in the hepatopancreas in treated vs. control groups. The antioxidant defense system can eliminate the ROS under non-stress conditions. However, excessive ROS are produced by chronic ammonia-N exposure, which inhibited the detoxification function, and other antioxidant enzymes may be needed to solve the problem. MDA is one of the major indicators reflecting the extent of oxidative damage [44]. The study showed that MDA content significantly increased with long-term ammonia-N exposure (*p* < 0.05), suggesting that the shrimp tissues were damaged by oxidative stress, while the results of the gill in treated vs. control groups indicated that there was no significant difference (*p* > 0.05). The increase in antioxidant enzyme activities, such as SOD, CAT, and MDA contents, suggested that the increased SOD and CAT activities were not sufficient for protection against stress-induced damage over a long period of time. It is commonly observed in many aquatic animals, such as rainbow trout (*Oncorhynchus mykiss*) [45], mangrove crab [46], and *Pinctada fucata* [47], that Na^+^/K^+^-ATPase (NKA) plays a significant role in the process of bronchial ammonia excretion. In the study, NKA activity in the two tissues significantly increased between the treated and control groups (*p* < 0.05). The results indicated that NKA participates in hepatopancreas- and gill-mediated NH4^+^ transport and that the latter is more sensitive to ammonia-N stimulation than the former. Similarly, the effects of chronic ammonia–N stress on juvenile ivory shell snails, *Babylonia areolata*, showed the same result [9]. Moreover, the histopathological variation in the two tissues showed trends of degeneration and necrosis, suggesting that chronic ammonia-N exposure may lead to tissue damage without recovery in *L*. *vannamei*.

### 4.3. Comparison Analysis between the Two Tissues

Transcriptome sequencing is an important tool for studying the mechanisms of growth, metabolism, immunity, etc. in non-model oganisms [48]. To better understand the molecular mechanism of chronic ammonia-N exposure, we carried out a comparative transcriptomics analysis between the hepatopancreas and gill groups. In the two groups, 62 overlapping DEGs were identified, indicating that the response to chronic ammonia-N stress varies significantly across the two tissues.

In the hepatopancreas in treated vs. control groups, DEGs were enriched in nine KEGG pathways, indicating that ammonia-N stress could affect carbohydrate metabolism, glycan biosynthesis, metabolism, and the immune system in the hepatopancreas of *L. vannamei*. Cellular stress and mutations in genes encode important factors and lead to reduced or abnormal ribosome production, which activates the tumor suppressor p53 and then reprograms cellular transcription [49]. Orsolic et al. reported that oxidative stress affects ribosome biogenesis [50]. The ribosome pathway was significant in this study (*p* < 0.05); most of the genes in it were down-regulated. In addition, dismutase (SOD) and catalase (CAT) are antioxidants that belong to the first line of defense antioxidants [51]. However, the study found that CAT was significantly up-regulated in the hepatopancreas in the treated vs. control groups (*p* < 0.05), whereas there was no significant difference in the gill in the treated vs. control groups (*p* > 0.05).

We speculate that the chronic ammonia-N stress may induce the oxidative stress and produce excess ROS and then restrain related genes’ expression. Moreover, the hepatopancreas was more sensitive to oxidative stress than the gills were. Proteasome and spliceosome pathways were also down-regulated after chronic ammonia-N stress (Table 4), which influenced the degradation of redundant and superfluous protein and metal RNA splices. The pentose and glucuronate interconversions pathway is involved in carbohydrate metabolism [52] and significantly enriched DEGs are related to detoxification [53]. This pathway is enriched after different types of stress, such as salinity stress [53], Zn exposure [33], and exposure to phoxim and prometryne residues in shrimp [54]. These pathways were significantly changed after ammonia-N exposure, indicating that it impacted protein biosynthesis and metabolism.

In the gill in treated vs. control groups, three KEGG pathways (mucin-type O-glycan biosynthesis, nucleocytoplasmic transport, and steroid biosynthesis) were significantly enriched and increased, indicating that ammonia-N stress could affect cellular homeostasis, biological processes, and protein function and structure in the gills of *L. vannamei*. The nuclear pore complex (NPC) is a kind of nuclear macro-complex that mediates nucleocytoplasmic transport (NCT). NPC plays an essential role in maintaining cellular homeostasis and influences transcription through interactions with chromatin. The depletion of NPC may lead to defects in mRNA transcription or nuclear output [55]. Morgan et al. reported that kras (G12V)-driven hepatocyte proliferation was prevented when the NCT inhibitor was administered to heterozygous ahctf1 larvae [56]. Some genes involved in NCT under ammonia-N stress conditions were up-regulated (*kpnb3*, *kidins220a*, *nup58*, *ran*, and *ipo9*) in this study (Table 4), which meant that the stress could increase gene transcription and protect cellular homeostasis. A previous study confirmed that *ipo9* coordinates the nuclear import of functionally related factors required for gametogenesis [57]. The *ran* overexpression significantly increased cellular tolerance to a variety of pesticides [58]. In addition, *ran* relocalization and nuclear transport were affected by stress treatment in animal cells [59]. Moreover, the expression of cytoskeleton-associated genes in the two tissues groups have significantly changed expression (*p* < 0.05). All genes were up-regulated in gill in treated vs. control groups in particular (Table 3). A previous study reported that the changes in cytoskeleton-associated genes were mostly likely to protect shrimp from ammonia-N exposure [60]. Additionally, the Na^+^/K^+^-ATPase, located on the basolateral membrane of gill cells, was associated with ammonia-N excretion in crustaceans, where K^+^ can be replaced by NH4^+^, which is excreted into the environment by the Na^+^/NH4^+^ exchanger [61]. Combining the findings with the MDA concentration and structural change, we hypothesize that gills adapted to chronic ammonia-N stress by excreting and activating relevant cytoskeleton/transcription gene expression to maintain cellular homeostasis. These results suggested that various tissues used different strategies or cooperated with each other in response to ammonia-N stress.

### 4.4. DEGs Involved in Lipids Metabolism and Serine Proteases

Cellular protection and detoxification pathways could enhance through environmental stress, leading to metabolic processes that require more energy consumption in aquatic animals [15]. In general, there are three indicators—protein, lipid, and glycogen reserves—for assessing the metabolic status of animals. Among these, protein and lipid contents are the two important indicators of evaluating temporal changes in available energy reserves in aquatic animals under stress [62]. However, three pathways involved in protein synthesis were down-regulated in the study, suggesting that chronic ammonia-N exposure affected the metabolic status of shrimp. Moreover, DEGs enriched in KEGG terms for ether lipid metabolism, sphingolipid metabolism, glycerophospholipid metabolism, and glycerolipid metabolism were found in both transcriptome analyses, with the expression of most genes showing an increasing trend (Table 5). When they are subjected and adapted to salinity stress, lipids are the energy source for fish [63]. Ammonia-N exposure may also cause oxidation and endoplasmic reticulum stress, leading to increased lipid synthesis and reduced lipid breakdown [64]. Therefore, we speculated that lipids were used to offer energy via metabolic pathways in response to ammonia-N stress. Additionally, serine-type endopeptidase activity, oxidoreductase activity, hydrolase activity, and serine hydrolase activity were GO terms enriched in DEGs from the two groups after chronic ammonia-N stress (Table 2). Sun et al. reported that they were connected to innate immunity in aquatic animals [65]. Interestingly, *ct55* is a novel and effective promoter of colitis-associated cancer (CAC) whose down-regulation indicates reduced inflammatory responses [66].

## 5. Conclusions

In this study, a comparative analysis between the hepatopancreas and gills of shrimp was performed to determine differences in growth performance, morphological and physiological alterations, and transcriptional responses under long-term ammonia-N exposure to probe the adaptation mechanism in *L. vannamei.* Chronic ammonia-N exposure significantly reduced growth, caused histological alterations, and changed the enzymatic activities in the two tissues. DEGs in the two tissues were enriched in pathways/genes associated with oxidative stress, protein synthesis, lipid metabolism, and different serine proteases. Moreover, the shrimp may offset ammonia-N toxicity at the transcriptome level by regulating gene expression. These results provided a new perspective in understanding the mechanisms of ammonia-N resistance in crustaceans.

## Figures and Tables

**Table 1:** Survival rate (SR), body length (BL), weight g.ain (WG), and specific growth rate (SGR) of *L. vannamei* exposed to ammonia nitrogen for 28 days.

Group	Survival Rate (%)	Body Length (mm)	Weight Gain (%)	Specific Growth Rate (% d^−1^)
Experimental	85.00 ± 1.00 ^a^	2.86 ± 0.28 ^a^	234.04 ± 0.50 ^a^	15.94 ± 0.01 ^a^
Control	89.17 ± 0.58 ^a^	3.97 ± 0.15 ^b^	416.99 ± 0.47 ^b^	25.31 ± 0.01 ^b^

Note: The corresponding data above are the mean ± standard deviation of two groups; the different superscript letters within the same column indicate significant differences (*p* < 0.05).

**Table 2:** Top 10 up- and down-regulated DEGs in hepatopancreas and gill groups.

Gene Names	log2FC	Description (KEGG)	Description (GO)
Hepatopancreas in treated vs. control groups			
*LOC113820612*	9.50639	chloroplastic-like	/
*LOC113807369*	6.40349	transmembrane protease serine 13-like	/
*LOC113803831*	5.52520	techylectin-5A-like	/
*ctrb1*	5.52161	chymotrypsin BII-like	serine-type endopeptidase activity (MF-up)
*LOC113802089*	5.46694	alcohol dehydrogenase [NADP(+)]-like	
*alpi.1*	5.36745	alkaline phosphatase-like	hydrolase activity, acting on ester bonds (MF-UP)
*tubb6*	5.24083	tubulin beta chain-like	hydrolase activity, acting on acid anhydrides (MF-Down)
*LOC113812240*	5.18027	methylenetetrahydrofolate reductase-like	/
*LOC113820103*	5.00419	mucin-12-like	/
*kcp*	4.96579	basic proline-rich protein-like	/
*LOC113802550*	−13.28640	triosephosphate isomerase B-like	/
*LOC113827511*	−12.04027	C-type lectin domain family 17, member A-like	/
*LOC113824334*	−8.99337	arylsulfatase B-like	/
*mmp3*	−8.66135	inactive pancreatic lipase-related protein 1-like	/
*LOC113807283*	−8.26976	ovochymase-2-like	/
*LOC113816677*	−7.90349	small integral membrane protein 8-like	/
*LOC113802551*	−7.60858	triosephosphate isomerase-like	/
*LOC113810108*	−7.00134	anti-lipopolysaccharide factor-like	/
*slc46a1*	−6.93143	solute carrier family 46-member 1	/
*mrpl2*	−6.76469	39S ribosomal protein L2, mitochondrial-like	ribonucleoprotein complex (CC-Down)
Gill in treated vs. control groups			
*wdr54*	8.28549	trypsin-1-like	serine hydrolase activity (MF-Down)
*LOC113819943*	8.23980	lactosylceramide 4-alpha-galactosyltransferase-like	/
*LOC113817714*	7.89112	glutamate-gated chloride channel subunit beta-like	/
*LOC113822681*	7.45779	large subunit ribosomal RNA	/
*hacd2*	7.41957	1-acyl-sn-glycerol-3-phosphate acyltransferase epsilon-like	transferase activity, transferring acyl groups (MF-UP)
*LOC113800217*	7.39103	CAP-Gly domain-containing linker protein 4-like	/
*LOC113819942*	7.27277	lactosylceramide 4-alpha-galactosyltransferase-like	/
*cyp27c1*	7.24919	probable cytochrome P450 49a1	oxidoreductase activity, acting on paired donors, with incorporation or reduction of molecular oxygen (MF-UP)
*LOC113823234*	7.09559	facilitated trehalose transporter Tret1-like	/
*fbxo32*	6.99950	F-box only protein 32-like	/
*LOC113820879*	−7.57815	galanin receptor type 1-like	/
*LOC113820814*	−6.84140	ETS-related transcription factor Elf-1-like	/
*znf143*	−6.46198	protein SpAN-like	endopeptidase activity (MF-Down)
*LOC113819193*	−6.27849	myosin heavy chain, muscle-like	/
*cs*	−6.15781	gastric triacylglycerol lipase-like	/
*LOC113830294*	−6.01273	cuticle protein 7-like	/
*LOC113818578*	−6.00421	histone-lysine N-methyltransferase, H3 lysine-79 specific-like	/
*LOC113830353*	−5.97509	extensin-like	/
*ct55*	−5.95853	trypsin-1-like	serine-type endopeptidase activity (MF-Down)
*LOC113830353*	−5.95598	carbohydrate sulfotransferase 10-like	/

**Table 3:** List of differentially expressed genes related to the cytoskeleton.

Genes	Hepatopancreas in Treated vs. Control Groups	Gills in Treated vs. Control Groups
paramyosin (*prm*)	/	up
tropomyosin (*tpm*)	down	/
flotillin (*flot*)	/	up
titin (*ttn*)	up	up
beta tubulin (*tub*)	down	up

**Table 4:** List of functional groups and related liver DEGs from hepatopancreas and gill groups in response to long-term ammonia-N exposure.

KEGGID	Funnation Description	Gene Names
Hepatopancreas in treated vs. control groups		
pvm03010	Ribosome	***LOC113826494***, *mrpl2*, *LOC113826493*, *LOC113822410*, *rsl24d11*, *rpl37*, *rpl7*, *rps27a*, *LOC113803764*, *rpl32*, *LOC113817813*, *LOC113822082*, *mrps6*, *LOC113812878*, *rpl29*, *rplp2*, *LOC113826804*, *LOC113817249*, *LOC113830328*, *LOC113827080*, *mrpl12*, *rpl36*, *rpl19*, *rps26*
pvm03050	Proteasome	*LOC113803698*, *psma4*, *psma1*, *psma2*, *psma3*, *psmd3*, *LOC113807430*, *psmb3*, *LOC113830026*
pvm03040	Spliceosome	*lsm8*, *snrpa1*, *LOC113820651*, *sf3b5*, *alyref*, *LOC113802307*, *snrpb*, *ppih*, *LOC113803628*, *snrpg*, *sf3a3*, *tra2b*, *LOC113823506*, *u2af1*, *sf3b6*, *snrpc*, *LOC113820949*, *lsm3*
pvm00670	One carbon pool by folate	***LOC113812240***, ***mtr***, ***shmt1***, *atic*, *dhfr*
pvm00531	Glycosaminoglycan degradation	*LOC113824334*, ***xpnpep2***, ***shisa7***, ***LOC113804791***, ***sgsh***
pvm01240	Biosynthesis of cofactors	***psat1***, ***alpi.1***, ***akr1b1.1***, ***LOC113802089***, *ugdh*, *LOC113826969*, ***shmt1***, *dhfr*, ***si:ch73-334d15.1***, *ak6*, ***LOC113828810***, *LOC113802542*, ***LOC113823260***, ***LOC113807674***, ***LOC113807126***, *coq7*
pvm00040	Pentose and glucuronate interconversions	***akr1b1.1***, ***LOC113802089***, *ugdh*, ***si:ch73-334d15.1***, ***sord***, ***asrgl1***
pvm00600	Sphingolipid metabolism	***cpxm1a***, ***xpnpep2***, *LOC113829078*, ***LOC113806775***, ***LOC113810646***, ***LOC113824094***, ***LOC113804791***, ***smpd1***
pvm03008	Ribosome biogenesis in eukaryotes	*LOC113826178*, *ak6*, *LOC113828897*, *LOC113816393*, *LOC113801028*, *LOC113826254*, *dkc1*, *LOC113816679*, *rrp7a*
Gill in treated vs. control groups		
pvm00512	Mucin type O-glycan biosynthesis	***galnt3***, ***plbd1***, ***LOC113806687***, ***LOC113806752***, ***LOC113801114***, *LOC113826876*, ***LOC113814026***
pvm03013	Nucleocytoplasmic transport	***LOC113821023***, ***kpnb3***, ***kidins220a***, ***nup58N***, ***ran***, ***LOC113814257***, ***LOC113811339***, ***LOC113822864***, *ipo7*, *nup153*, ***ipo9***, *LOC113807607*
pvm00100	Steroid biosynthesis	***LOC113822972***, ***LOC113810916***, *cs*

Note: the bold indicates the up-regulated genes, and non-bold indicates down-regulated genes in these pathways.

**Table 5:** The related genes in the lipid metabolism pathway.

KEGGID	Funnation Description	Gene Names
LA vs. LAC		
pvm00565	Ether lipid metabolism	*LOC113829078*
pvm00600	Sphingolipid metabolism	***cpxm1a***, ***xpnpep2***, *LOC113829078*, ***LOC113806775***, ***LOC113810646***, ***LOC113824094***, ***LOC113804791***, ***smpd1***
pvm00564	Glycerophospholipid metabolism	***LOC113808891***, ***LOC113810367***, ***PCYT2***, ***LOC113802189***, ***LOC113805268***, ***LOC113814898***
pvm00561	Glycerolipid metabolism	***akr1b1.1***, ***LOC113802089***
LB vs. LBC		
pvm00565	Ether lipid metabolism	***agps***, ***LOC113821399***, ***LOC113824844***
pvm00600	Sphingolipid metabolism	***gba***, ***asah1***
pvm00564	Glycerophospholipid metabolism	***hacd2***, ***LOC113821399***
pvm00561	Glycerolipid metabolism	***LOC113823713***, ***hacd2***, ***LOC113819701***, ***LOC113817093***

Note: the bold indicates the genes up-regulated; non-bold indicates down-regulated genes in these pathways.

## Data Availability

Data will be made available on request.

## Supplementary Materials

The following supporting information can be downloaded at: https://www.mdpi.com/article/10.3390/ani13243799/s1, Table S1: Range of water quality-related indicators; Table S2: Survival time of the shrimp; Table S3: Primer sequences used in this study; Table S4: Assembly statistic of *L. vannamei* transcriptome; Table S5: Compare statistics with reference genome.
