# Supplementary material for: Differential Toxicity Responses between Hepatopancreas and Gills in Litopenaeus vannamei under Chronic Ammonia-N Exposure
Source: Animals (Basel). 2023 Dec 9;13(24):3799. doi: 10.3390/ani13243799 (PMC10741007; doi:10.3390/ani13243799)
Supplement: Supplementary file 1 [file animals-13-03799-s001.zip › animals-2690047-supplementary.pdf]

Supplementary material

Table S1 Range of water quality-related indicators

Group	Temperature (°C)	pH	Salinity (ppt)	Ammonia-N concentration (mg/L)
Experimental	28–30	7.8–8.3	30–31	7.6–8.2
Control	28–30	7.9–8.2	30–31	0–0.25

Table S2 Survival time of the shrimp

Time or item	Treated groups (time)			Control groups (time)		
	T1	T2	T3	C1	C2	C3
2nd day	1 (11:00 am)	/	/	/	/	/
3rd day	/	1 (7:00 am)	/	/	/	/
5th day	/	/	1 (16:00 pm)<br>1 (23:00 pm)	/	1 (16:00 pm)	1 (16:00 pm)
6th day	2 (7:00 am)	/	1 (16:00 pm)	1 (7:00 am)	/	/
7th day	/	1 (16:00 pm)	/	/	/	/
8th day	/	1 (11:00 am)	/	/	/	/
9th day	/	/	/	/	/	/
10th day	/	/	/	1 (23:00 pm)	/	/
11th day	/	/	/	/	/	/
12th day	/	/	/	/	1 (23:00 pm)	/
14th day	/	/	/	/	/	1 (7:00 am)
15th day	/	1 (23:00 pm)	2 (7:00 am)	/	/	/
17th day	1 (11:00 am)	/	/	1 (7:00 am)	/	/
19th day	1 (16:00 pm)	/	/	/	1 (16:00 pm)	/
20th day	/	/	1 (23:00 pm)	/	1 (16:00 pm)	/
21th day	/	1 (11:00 am)	/	/	/	/
22th day	/	/	/	1 (11:00 am)	1 (7:00 am)	/
23th day	/	1 (16:00 pm)	1 (16:00 pm)	/	/	/
26th day	/	/	/	/	/	2 (11:00 am)
Cumulative number	5	6	7	4	5	4

Table S3 Primer sequences used in this study

Gene	Gene ID	Primer name	Primer sequence (5’-3’)	Purpose
β-actin	LOC113813020	*β-actin*-F	5’-CCCTCGCTCCCTCCACCATG	Reference
		β-actin-R	5’-CTCCTGCTTGCTGATCCACATCTG	gene
dnajc5	LOC113802295	*dnajc5*-F	5’-GGAAGCGACTACGGCAAGA	
		dnajc5-R	5’-TAAGCCACCCAGGCAGAATA	
lpla2	LOC113805268	*lpla2*-F	5’-TGTTGAGACGCCCACGAA	
		lpla2-R	5’-TTGCCAGCAGCATAGACCA	
slc6a8	LOC113816986	*slc6a8*-F	5’-TTGACTTGTTCCCCGACTACC	
		slc6a8-R	5’-GGATGATACCGCTGGCTGA	
titin	LOC113819544	*titin*-F	5’-ACCGTAACCTTGAAATGCTGAT	
		titin-R	5’-TTCTTGCCACCTCCTCTGC	qPCR
ferritin	LOC113830150	*ferritin*-F	5’-TTTGCCCAGGACACCGTAG	
		ferritin-R	5’-GACACCTCGCTTGTTGAGATAGT	
naa38	LOC113808996	*naa38*-F	5’-CTCGTGGTCTGCCTGGTCTT	
		naa38-R	5’-AGGTCTGAGCGGTGGTCTTC	
sec61b	LOC113809401	*sec61b*-F	5’-GCAAGACCACCACCTCTACCT	
		sec61b-R	5’-CAACTCCACAGCGTCACTCG	
pck2	LOC113826096	*pck2*-F	5’-TCAGGCTATGGCGGAAACT	
		pck2-R	5’-GGGAAGGCAGCAGCAATGTA	

Table S4 Assembly statistic of *L. vannamei* transcriptome

Groups	Total raw reads	Total clean reads	Clean bases	GC Percentage	Q20%	Q30%
Hepatopancreas in treated vs. control groups						
Experimental-1	42472940	41962000	6.29G	48.49	97.11	92.29
Experimental-2	50645028	49716510	7.46G	49.09	97.96	94.02
Experimental-3	46534620	45145328	6.77G	49.06	97.88	93.85
Control-1	43280490	42364064	6.35G	48.60	97.95	94.01
Control-2	49168174	47505864	7.13G	47.32	97.37	92.84
Control-3	40328882	39518692	5.93G	49.22	97.58	93.35
Gill in treated vs. control groups						
Experimental-1	42601400	41498544	6.22G	42.79	97.91	93.91
Experimental-2	47370314	46295474	6.94G	44.50	97.49	92.86
Experimental-3	40609918	39577796	5.94G	43.90	97.77	93.72
Control-1	40138518	37237092	5.59G	41.10	95.08	88.89
Control-2	49709960	48913838	7.34G	43.78	97.21	92.55
Control-3	46721526	45406294	6.81G	39.16	95.47	89.52

Table S5 Compare statistics with reference genome

Groups	Effective reads	Total mapped	Exon	Intron	Intergenic
Hepatopancreas in treated vs. control groups					
Experimental-1	41962000	37594435 (89.59%)	5207381562 (92.76%)	110603290 (1.97%)	295936998 (5.27%)
Experimental-2	49716510	44692149 (89.89%)	6151948441 (92.17%)	152510703 (2.29%)	369790528 (5.54%)
Experimental-3	45145328	40525038 (89.77%)	5559714483 (91.87%)	150740605 (2.49%)	341082494 (5.64%)
Control-1	42364064	38101540 (89.94%)	5217829921 (91.69%)	126047855 (2.21%)	346993398 (6.10%)
Control-2	47505864	41985312 (88.38%)	5633428419 (89.88%)	102937280 (1.64%)	531312967 (8.48%)
Control-3	39518692	33903074 (85.79%)	4604517224 (91.02%)	98385461 (1.94%)	355838687 (7.03%)
Gill in treated vs. control groups					
Experimental-1	41498544	37318272 (89.93%)	4822262831 (86.54%)	144763576 (2.60%)	605114407 (10.85%)
Experimental-2	46295474	42181381 (91.11%)	5587102513 (88.68%)	185118931 (2.94%)	528253883 (8.38%)
Experimental-3	39577796	35363980 (89.35%)	4540594922 (86.00%)	168238449 (3.19%)	570899306 (10.81%)
Control-1	37237092	27725198 (74.46%)	3092863223 (74.94%)	124681921 (3.02%)	909588327 (22.04%)
Control-2	48913838	42769779 (87.44%)	5245687342 (82.19%)	215967293 (3.38%)	920506174 (14.42%)
Control-3	45406294	34032831 (74.95%)	3750937527 (74.01%)	157132678 (3.10%)	1160072907 (22.89%)
